# Effectiveness of perioperative remimazolam in preventing postoperative delirium: a systematic review and meta-analysis

**DOI:** 10.1186/s40001-025-02383-z

**Published:** 2025-02-21

**Authors:** Mingzhen Wang, Jinhui Liu, Wenjie Liu, Xin Zhang, Gaofeng Zhang, Lixin Sun, Yanlin Bi, Hong Wang, Rui Dong

**Affiliations:** 1 School of Anesthesiology, Shandong Second Medical University, No. 5 Donghai Middle Road, Qingdao, 266071 China; 2 Department of Anesthesiology, Qingdao Hospital, University of Health and Rehabilitation Sciences (Qingdao Municipal Hospital), Qingdao, China; 3 Department of Anesthesiology, Dezhou Third People’S Hospital, Dezhou, China; 4 Department of Pediatrics, Qingdao Women and Children’S Hospital, Qingdao University, No. 217 Liaoyang West Road, Qingdao, 266011 China; 5 Key Laboratory of Anesthesiology and Resuscitation, Huazhong University of Science and Technology), Ministry of Education, Wuhan, China

**Keywords:** Postoperative delirium, Remimazolam, General anesthesia, Safety, Meta-analysis

## Abstract

**Background:**

To compare the POD rates in patients undergoing non-cardiac surgery who received remimazolam perioperatively versus placebo or other sedatives.

**Methods:**

We systematically searched four major databases (Cochrane Central Register of Controlled Trials, Web of Science, Embase, and PubMed) for relevant randomized controlled trials (RCTs) up to July 11, 2024. Literature quality evaluation was used the bias risk table in Review Manager 5.4. The primary outcome of interest was POD, and secondary outcomes were the hypotension risk, bradycardia and, nausea and vomiting.

**Results:**

Across 11 trials involving 1985 participants, we recorded 309 cases of POD during follow-up. In trials where the control group received saline, remimazolam decrease the risk of POD significantly by 70% (RR 0.30, 95% CI [0.19, 0.46]; *p* < 0.00001). Statistical analysis did not show significant difference in the risk of POD between the remimazolam group and the groups receiving either dexmedetomidine (RR 1.23 [0.64, 2.37]; *p* = 0.53) or propofol (RR 0.83 [0.60, 1.16]; *p* = 0.28). Regarding adverse events, remimazolam significantly reduces the morbidity of hypotension compared to dexmedetomidine (RR 0.25 [0.10, 0.65]; *p* = 0.004) and propofol (RR 0.45 [0.33, 0.60]; *p* < 0.00001). In addition, there were no significant differences in the incidence of bradycardia (RR 0.85; 95% CI [0.34–2.12], *p* = 0.72) and nausea and vomiting (RR 1.06; 95% CI [0.74–1.51], *p* = 0.77) between remimazolam and the control group.

**Conclusions:**

During the perioperative period, using remimazolam can lower POD risk after surgery for patients who had non-cardiac surgery, but remimazolam does not work better than dexmedetomidine or propofol. Compared with the dexmedetomidine and propofol, remimazolam also has apparent advantages in preventing intraoperative hypotension.

## Introduction

Postoperative delirium (POD) is a complication that often occurs in the early postoperative period and is characterized by a decline in cognitive function, such as confusion or disorientation. POD can manifest as hyperactivity, hypoactivity, or mixed states, ultimately contributing to adverse events, such as increased complications, delayed discharge, long-term cognitive impairment, and increased mortality [1, 2]. POD can occur at all ages, but it is more common among the elderly population. Due to the complex etiology, prophylactic or curative treatments for POD are often ineffective [3]. In addition, the risk of POD increases significantly with age, especially in half of patients 65 years and older who develop delirium after surgery [4]. Studies have shown that POD affects approximately one-third of general patients aged 70 and above [5]. Many intraoperative factors can result in more POD in postoperative patients, such as the need for blood transfusion, fluid administration, and the trauma from surgery [6]. Timely identification of possible risks to patients before surgery can effectively reduce the POD risk [7], while multifaceted interventions, including early diagnosis and prompt treatment, medication review, pain management, and preventing complications, are the best option for POD treatment, the effective implementation of POD monitoring [8], treatment and precautions remains a significant obstacle for healthcare organizations globally [9].

Remimazolam is a water-soluble, ultra-short-acting benzodiazepine with a structure similar to midazolam. It exerts its sedation by enhancing the activity of gamma-aminobutyric acid type A receptors, leading to membrane hyperpolarization and an increase in the chloride ion influx, ultimately inhibiting neuronal activity [10]. Compared to other benzodiazepines, remimazolam has diverse distinct pharmacological properties, including a fast onset of action, independent of organ metabolism [11], short duration of action, predictable recovery and controllable sedation [12], reversibility, and good hemodynamic stability. These characteristics suggest that remimazolam may have more advantages over short-acting sedatives in specific clinical environments [13]. Remimazolam has shown a promising perspective, but its impacts on postoperative outcomes in non-cardiac surgery patients still need to be determined. There is also less discussion about whether remimazolam can reduce POD compared with other anesthetic sedatives. A non-inferiority trial involving 728 non-cardiac surgery patients found that there is no significant difference in POD risk between remimazolam and propofol [14]. However, a randomized controlled trial conducted by Duan et al. on the same surgical type demonstrated a significant reduction in POD when using remimazolam [15].

At present, more and more new studies provide much data on the effect of remimazolam on POD, but whether benzodiazepines can reduce POD is still controversial [16, 17]. We used 12 sets of data from 11 trials to conduct a meta-analysis aimed at comparing the effects of remimazolam and placebo (or other sedative agents) on the risk of POD, thereby providing more accurate evidence for the selection of rational anesthesia sedative agents to prevent the occurrence of POD in clinical practice.

## Methods

### Search strategy

This study was conducted in accordance with the PRISMA guidelines to ensure transparency and rigor [18]. It was also registered in the PROSPERO database (CRD42024567693), thereby promoting reproducibility and future research.

We thoroughly searched four major databases (Cochrane Central Register of Controlled Trials, Web of Science, Embase, and PubMed) to identify all relevant studies published from the inception of these databases up to July 11, 2024. We use a combination of Medical Subject Headings (MeSH) and free words to build a search strategy. The primary search strategy employed in PubMed is outlined below: ("delirium" [MeSH] OR “delirium” [All Fields] OR “postoperative delirium” [All Fields] OR “postoperative cognitive decline” [All Fields] OR POCD [All Fields] OR “early postoperative cognitive dysfunction” [All Fields] OR “postoperative cognitive dysfunction [All Fields]”) AND (“remimazolam” [All Fields]). Only randomized controlled trials (RCTs) were included in the search. There are no restrictions on the publication language. Furthermore, we examined relevant reference lists and searched trial registries and registration agencies to conduct a comprehensive search to ensure no relevant studies were omitted in the initial database searches.

### Study selection criteria

This meta-analysis included pediatric, adult male, and female patients aged 3 years and older who underwent non-cardiac surgery. The inclusion criteria were restricted to published, full-text RCTs that compared the efficacy of perioperative remimazolam with placebo in preventing POD following non-cardiac surgery.

### Data extraction

Data extraction was performed independently by two authors (M. Wang and J. Liu) using a standardized data collection form. Discrepancies were resolved through discussion with two reviewers (R. Dong and L. Sun). Extracted data included the following variables: first author’s name, the year of publication, type of surgery, remimazolam loading dose and infusion rate, control group type, control group loading dose, and control group infusion rate.

### Assessment of trial quality

To assess the methodological quality of the included trials, three authors (H. Wang, W. Liu and X. Zhang) independently evaluated the bias risk in the included literature using the risk of bias table in Review Manager 5.4. This evaluation covers six areas and aims to assess potential bias. First, the methods used to generate the random sequence and conceal allocation were assessed to determine whether there was selection bias. Second, whether participants and researchers were blinded to treatment allocation was assessed to assess implementation bias. Third, whether outcome assessors were blinded to treatment allocation was assessed to assess detection bias. Fourth, the completeness of outcome data and whether there was differential loss to follow-up between treatment groups was assessed to assess dropout bias. Fifth, whether there was selective reporting of outcomes was assessed to assess reporting bias. Finally, other potential biases that could have affected the study results were considered. Each domain was divided into three risk levels: low risk, unclear risk, or high risk. Any discrepancies in this assessment were resolved through discussion with two researchers (G. Zhang and Y. Bi).

### Statistical analysis

Review Manager software (RevMan version 5.4) was used to perform statistical analysis. We analyzed dichotomous data, specifically the occurrence of POD, and calculated risk ratios (RRs) with 95% confidence intervals (CI). The I^2^ statistic calculated in Review Manager, was used to assess heterogeneity. Heterogeneity was considered low (*I*^2^ < 50%), moderate (*I*^2^ = 50–75%), and high (*I*^2^ > 75%) [19]. Data were pooled using a fixed-effect model when I^2^ was less than 50%; otherwise, we used a random-effects model. Three pre-specified subgroup analyses were conducted based on the control group type (saline, dexmedetomidine, or propofol). We performed sensitivity analysis to assess the robustness of the results. This involved sequentially omitting one study at a time, and evaluating the influence of individual studies on the overall effect size. Finally, statistical significance for interpreting the results was set at a *p* < 0.05.

## Results

### Search results

**Fig. 1:**
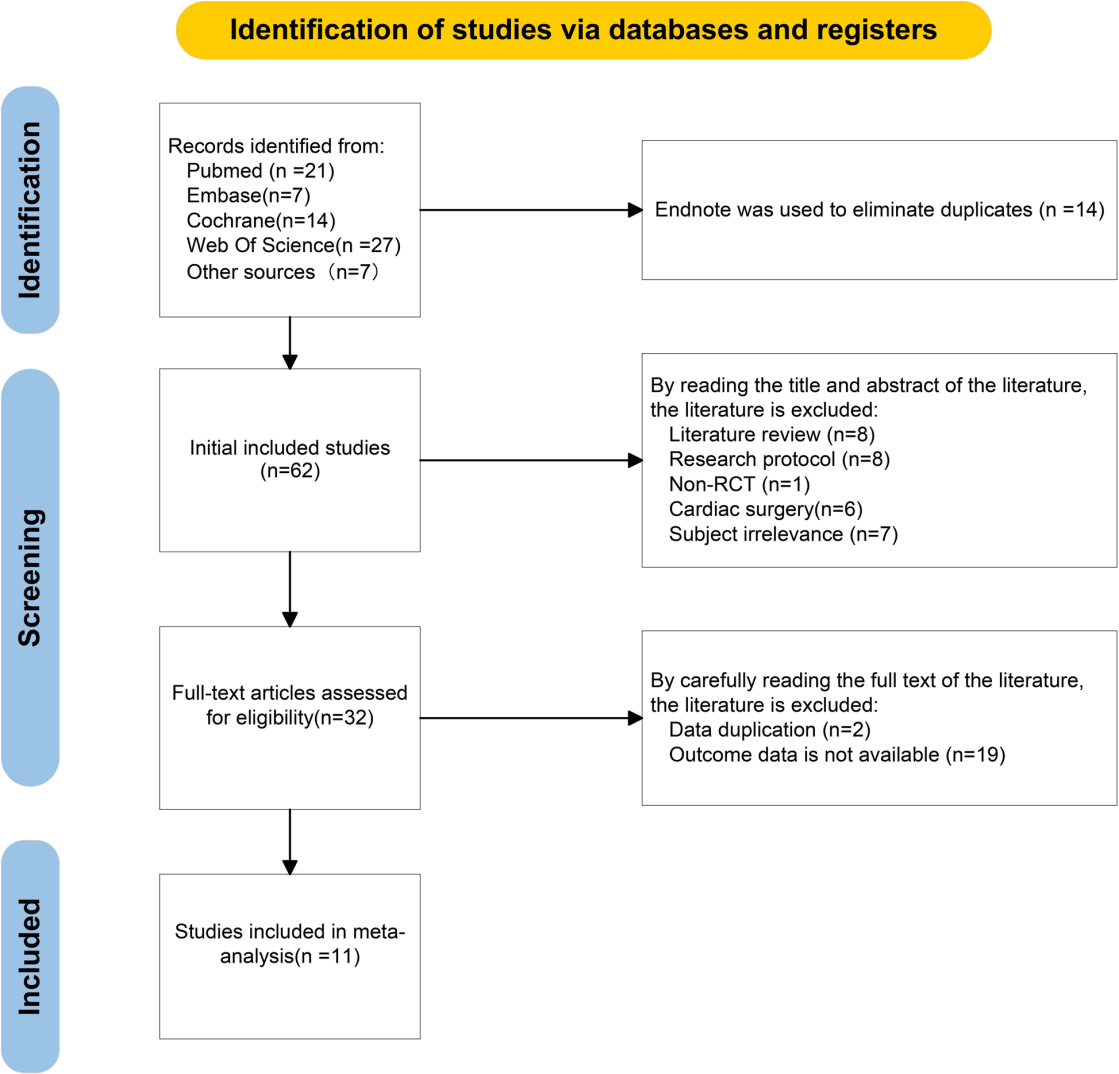
PRISMA flow diagram of the literature search process

To identify potentially eligible studies, a systematic literature search was conducted in the Cochrane Library, Web of Science, EMBASE, and PubMed. Nine duplicate records were excluded after a rigorous screening process based on author names, publication dates, and journal titles. Further review of titles and abstracts resulted in the exclusion of 31 studies. Next, we conducted a full-text review of the remaining 13 literatures, identifying 11 RCTs [11, 12, 14, 15, 20–26] that met the predefined inclusion criteria and were included in the meta-analysis. Including the reasons for exclusion, the study selection process is illustrated in the flow diagram (Fig. 1).

### Characteristics of trials

This meta-analysis included 11 trials published between 2022 and 2024, with 1985 patients (1055 in the remimazolam group and 930 in the placebo group). All trials were published in English. Table 1 provides a comprehensive overview of the characteristics of the included trials.

**Table 1:** Study characteristics of the included studies

The first author, year	Age (years)	Surgery type	No. of patients		Remimazolam		Control			Timing of infusion
			Remimazolam	Control	Loading dose	Infusion rate	Type	Loading dose	Infusion rate	
Cai, 2024 (1)	1‐6	Laparoscopic surgery	40	40	No	1 mg/kg/h	Saline	No	1 mL/kg/h	Before anesthesia
Cai, 2024 (2)	1‐6	Laparoscopic surgery	39	40	0.2 mg/kg	No	Saline	No	1 mL/kg/h	Before anesthesia
Liao, 2023 (2)	65–80	Laparoscopic radical resection of gastric cancer	34	35	0.2 mg/kg	0.3–0.5 mg/kg/h	Saline	No	0.3–0.5 mL/kg/h	Before anesthesia
Yang X, 2022	3–7	Tonsillectomy and adenoidectomy	51	50	0.2 mg/kg	Not described	Saline	No	Not described	Before anesthesia
Deng, 2022	> 70	Orthopedic surgery	37	38	0.075 mg/kg	0.1–0.3 mg/kg/h	Dexmedetomidine	0.5 μg/kg	0.2–0.7 μg/kg/h	After anesthesia
Liao, 2023(1)	65–80	Laparoscopic radical resection of gastric cancer	34	35	0.2 mg/kg	0.3–0.5 mg/kg/h	Dexmedetomidine	0.5 μg/kg	0.3–0.5 μg/kg/h	Before anesthesia
Duan, 2023	60–75	Hip replacement	30	30	0.2–0.4 mg/kg	0.3–0.5 mg/kg/h	Propofol	1.5–2 mg/kg	4–8 mg/kg/h	Before anesthesia
Fang, 2024	60–90	Hip surgery	364	364	0.2–0.25 mg/kg	Not described	Propofol	1.5–2 mg/kg	Not described	Before anesthesia
Liu, 2024	≥ 65	Radical resection of colon cancer	50	50	0.1–0.2 mg/kg	0.4–1.2 mg/kg/h	Propofol	1–2 mg/kg	4–10 mg/kg/h	Before anesthesia
Luo, 2023(L)^a^	18–60	Short laparoscopic surgery	47	49	6.0 mg/kg/h	1 mg/kg/h	Propofol	2.0 mg/kg	6.0 mg/kg/h	Before anesthesia
Luo, 2023(M)^b^	18–60	Short laparoscopic surgery	48	49	9.0 mg/kg/h	2 mg/kg/h	Propofol	2.0 mg/kg	6.0 mg/kg/h	Before anesthesia
Luo, 2023(H)^c^	18–60	Short laparoscopic surgery	48	49	12.0 mg/kg/h	3 mg/kg/h	Propofol	2.0 mg/kg	6.0 mg/kg/h	Before anesthesia
Pan, 2022	> 18	Rigid bronchoscopy	15	15	0.4 mg/kg	1 mg/kg/h	Propofol	1.5 mg/kg	4–8 mg/kg/h	Before anesthesia
Yang, 2023	> 60	Orthopedic surgery	147	153	0.2–0.3 mg/kg	Not described	Propofol	1.0–1.5 mg/kg	Not described	Before anesthesia
Zhang, 2024	≥ 18	Cerebral endovascular procedures	71	71	0.1 mg/kg	0.3–0.7 mg/kg/h	Propofol	1.0–1.5 mg/kg	4–10 mg/kg/h	Before anesthesia

^a^L means low group which use low loading dose and infusion rate; ^b^M means median group which use median loading dose and infusion rate; ^c^H means high group which use high loading dose and infusion rate

### Risk of bias in included studies

**Fig. 2:**
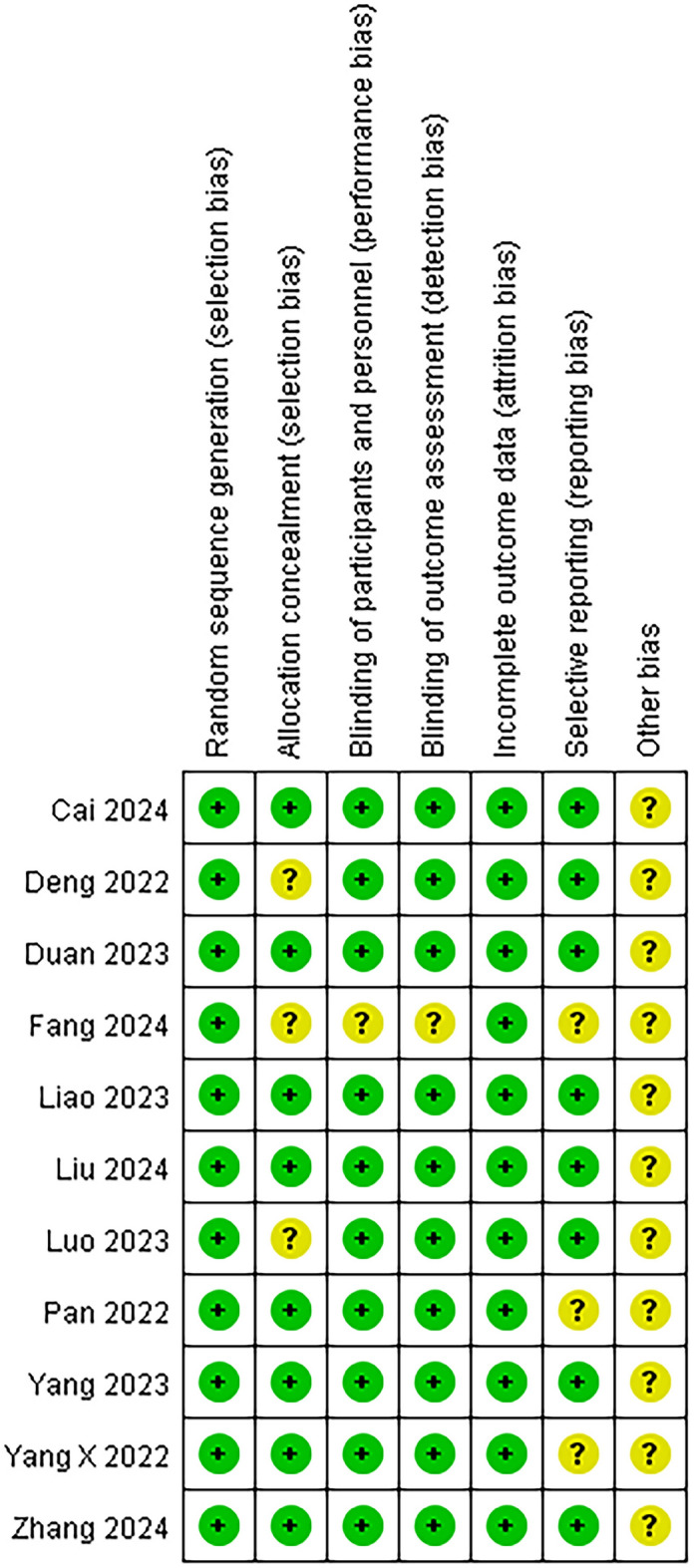
Methodological quality of trials using the Cochrane risk of bias methods. ( +), low risk of bias; (?), unclear; ( −), high risk of bias

We used the risk of bias table in Review Manager 5.4 to assess the quality of the 11 included experiments, the overall risk of bias in the included literature was low, and there were no items with a significantly high risk of bias. The details of the assessment are shown in Fig. 2.

### Effect of interventions

#### Primary outcomes

**Fig. 3:**
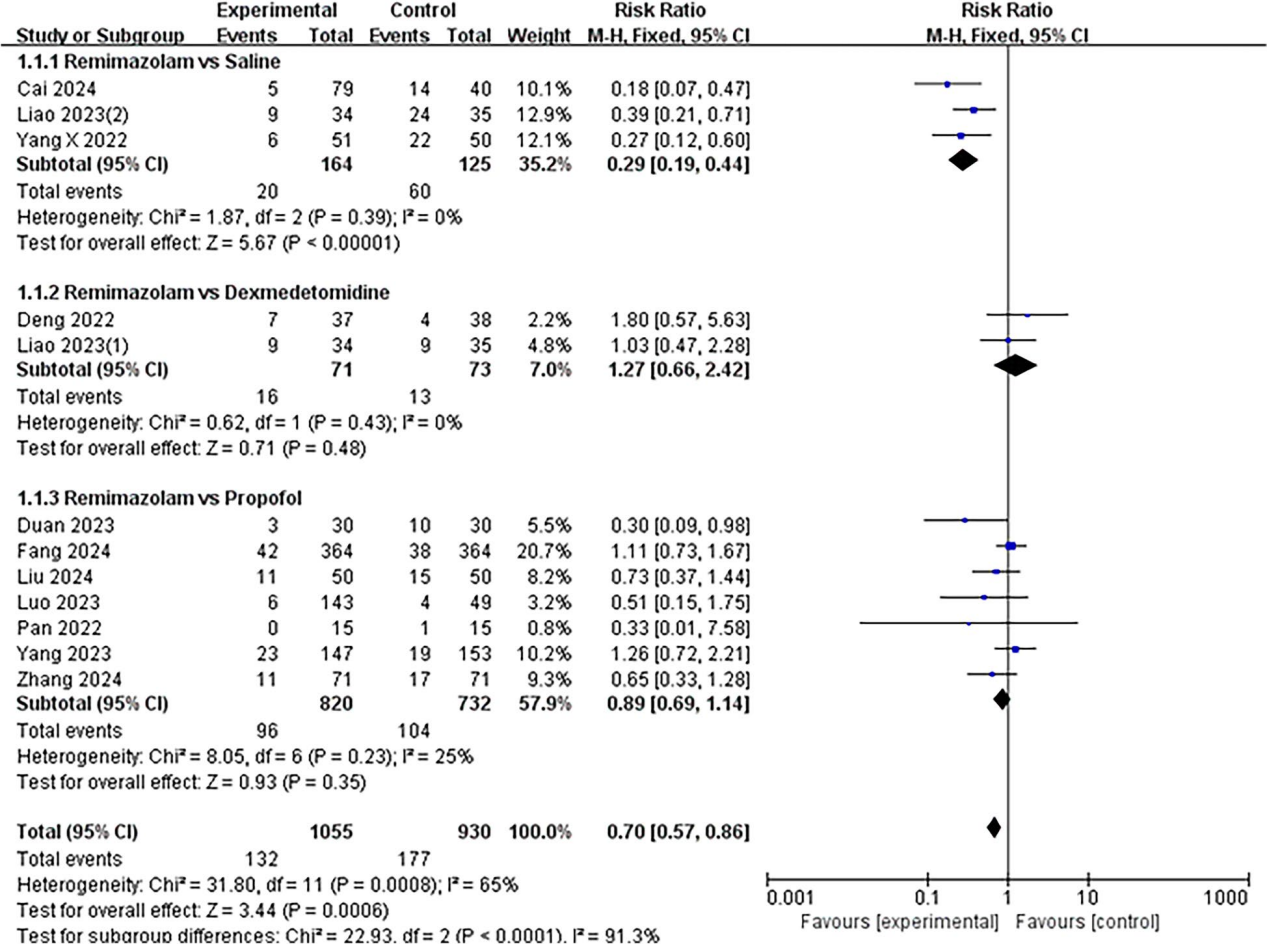
Outcome of postoperative delirium after remimazolam sedation versus placebo (or other sedation) sedation

Among the 1985 patients included, 309 experienced POD. The analysis revealed that remimazolam exhibited a significant reduction in the incidence of POD following non-cardiac surgery compared to saline across three trials (RR 0.30, 95% CI [0.19, 0.46]; *p* < 0.00001). Despite the significant reduction in POD incidence observed with remimazolam compared to saline, no statistically significant difference was found when comparing remimazolam to dexmedetomidine or propofol control groups. Two trials included dexmedetomidine as a control (RR 1.23; 95% CI [0.64–2.37], *p* = 0.53, *I*^2^ = 0%), while seven trials used propofol as a control (RR 0.83; 95% CI [0.60–1.16], *p* = 0.28, *I*^2^ = 25%) (Fig. 3).

#### Secondary outcomes

**Fig. 4:**
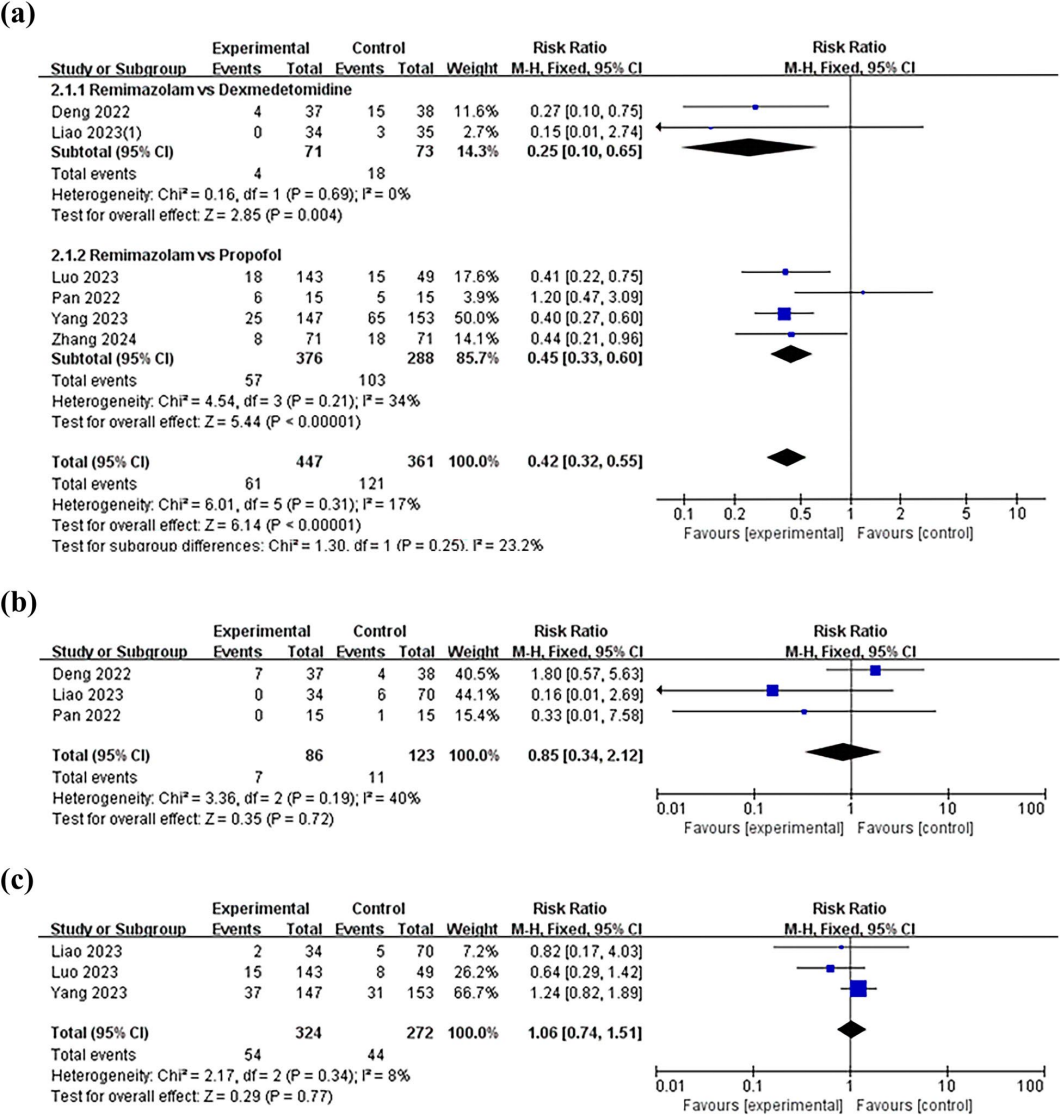
**a** Forest plot of intraoperative hypotension. **b** Forest plot of intraoperative bradycardia. **c** Forest plot of nausea and vomiting

Intraoperative hypotensive events were reported in 978 patients included in the seven experimental groups. However, the data from two of the groups with saline as the control group were insufficient to be analyzed due to imperfections, so after subgroup analyses with dexmedetomidine or propofol as the control group, the findings demonstrated that remimazolam reduces the incidence of intraoperative hypotension compared with either dexmedetomidine(RR 0.25; 95% CI [0.10–0.65], *p* = 0.004, *I*^2^ = 0%) or propofol(RR 0.45; 95% CI [0.33–0.60], *p* < 0.00001, *I*^2^ = 34%) (Fig. 4a). In three trials (*n* = 209), the risk of bradycardia with remimazolam was not statistically different from the control (RR 0.85; 95% CI [0.34–2.12], *p* = 0.72, *I*^2^ = 40%) (Fig. 4b). Similarly, an analysis of nausea and vomiting data from three independent trials (*N* = 596) demonstrated that remimazolam did not significantly alter the risk of these events compared to the control group (RR 1.06; 95% CI [0.74–1.51], *p* = 0.77, *I*^2^ = 8%) (Fig. 4c).

### Sensitivity analysis and publication bias

**Fig. 5:**
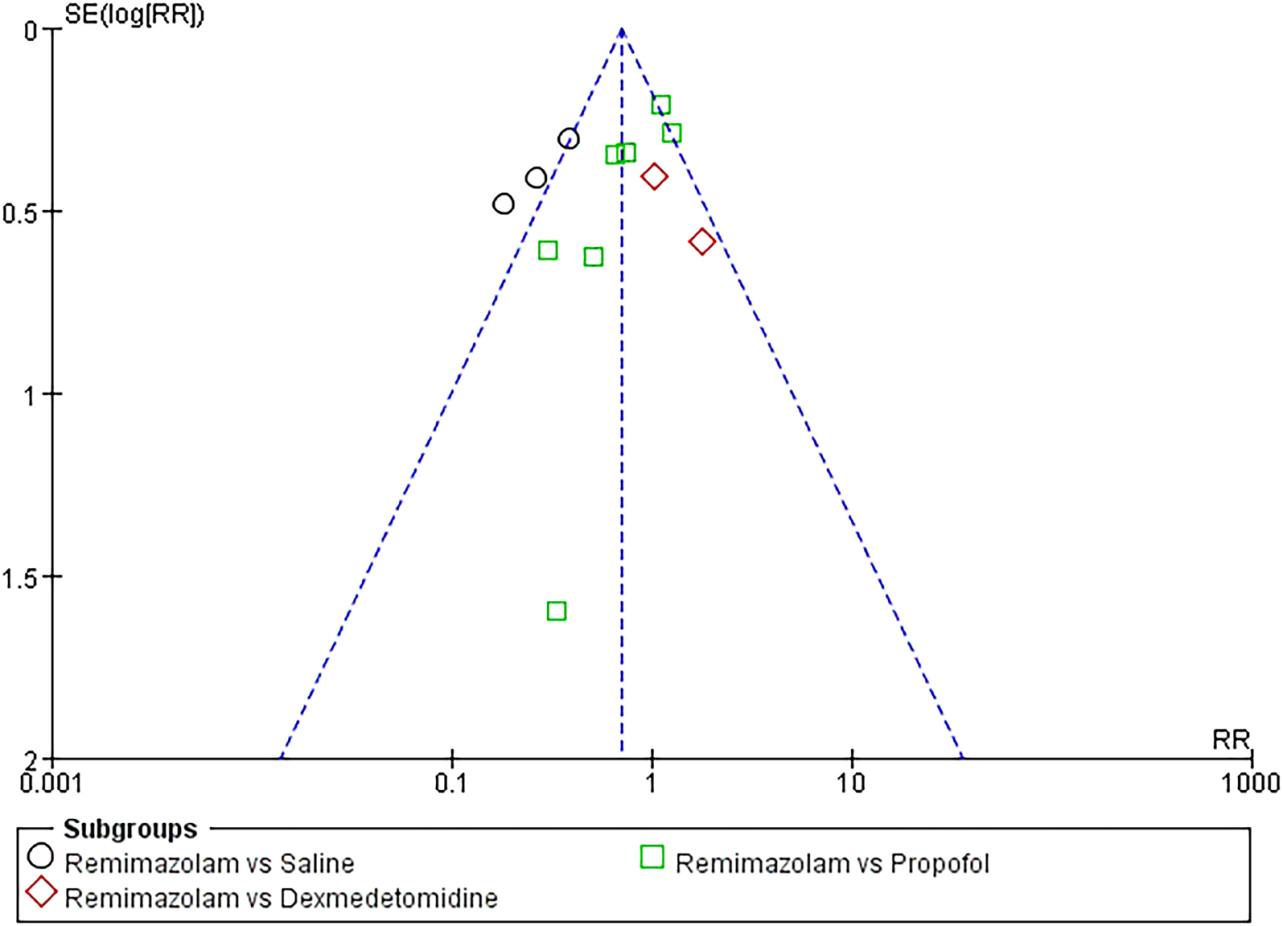
Funnel plot of the primary outcome (postoperative delirium after non-cardiac surgery)

We conducted a sensitivity analysis to evaluate the robustness of the meta-analysis findings by systematically excluding individual studies from the analysis. The results show that the final results are consistent with previous conclusions, suggesting that the final results are not caused by any single study. Publication bias was assessed through visual inspection of funnel plots. The symmetry of the funnel plots indicated no evidence of publication bias regarding the incidence of POD outcome (Fig. 5).

## Discussion

This meta-analysis compared remimazolam with placebo (or other sedatives), suggesting that remimazolam significantly reduced the POD risk in patients after non-cardiac surgery.

POD is a severe condition whose risk is increased by factors, such as elevated stress levels and the use of certain drugs. We must recognize the possibility of POD in patients and timely identify and address the risk factors. To relieve these risks, using a multi-modal approach that includes prevention strategies and interventions for POD patients is critical [27]. Little is known about the pathophysiological mechanisms underlying POD, but the most common explanations include neurotransmitter imbalances and neuroinflammation [5]. POD is typically attributed to a confluence of diverse etiological factors, such as surgery, using potentially pro-inflammatory drugs, and the hospital ward environment, which includes underlying medical conditions and prescription drugs. Due to the complexity of POD’s etiology, its pathophysiology and psychopathology are similarly complex and variable [28]. While sedation is essential for mechanical ventilation during anesthesia, they have also been related to the development of POD [29]. Previous studies have shown that a large percentage of patients on mechanical ventilation (between 60 and 80%) experience POD [30–32].

The exact mechanism by which remimazolam reduces the incidence of POD remains to be fully elucidated. Current research indicates its potential protective effects may be attributed to a multi-factor approach, such as sedation, gamma-aminobutyric acid (GABA) receptor agonism, anti-inflammatory capacity, vasodilation, and antioxidant activity. Remimazolam can enhance the inhibitory neurotransmission of GABA receptors, thus alleviating neuron hyperexcitability during surgery and potentially contributing to neuroprotection [33]. Remimazolam can reduce postoperative inflammatory response by inhibiting the release of inflammatory mediators [34], and this effect may play a role in reducing the risk of postoperative cognitive dysfunction and POD [21, 35]. Remimazolam may also have a slight effect of vasodilation [36, 37], which could improve the blood flow to the brain and increase cerebral oxygenation. This might help to reduce the risk of cerebral hypoxia–ischemia. Its sedation may further improve the sleep quality of postoperative patients and promote brain recovery. The antioxidant effects of remimazolam help protect neurons from free radical damage and improve postoperative cognitive function by affecting neuronal plasticity, such as the regulation of nerve growth factor [38].

Compared to propofol, remimazolam’s sedative effects are readily reversed by the specific antagonist flumazenil, which exhibits more favorable hemodynamic characteristics and has minimal inhibitory effects on the heart [39, 40]. Remimazolam’s rapid onset of action and rapid metabolism, along with its good safety profile, make it a more and more appealing option for use as an intravenous anesthetic in a broad range of clinical applications. The comparison of remimazolam with saline in this meta-analysis also demonstrated its effect in reducing POD. Despite the study’s lack of statistically significant findings regarding POD reduction, between remimazolam and dexmedetomidine or propofol, the findings still have reference significance in the comprehensive selection of anesthetics. This lack of significant difference does not necessarily indicate a lack of effect, but could be influenced by several factors. First, the relatively small sample sizes in the included studies comparing remimazolam with dexmedetomidine or propofol may have reduced the power to detect smaller, clinically relevant differences, increasing the risk of Type II errors. For instance, the study by Duan et al. which found no reduction in POD with propofol, had a smaller sample size compared to studies included in our saline comparison [28]. In addition, there was significant heterogeneity in the patient populations across studies. Some study included primarily elderly patients with multiple comorbidities undergoing high-risk surgeries, such as radical resection of colon cancer, which could have a higher baseline risk of POD regardless of the choice of anesthetic [22]. These baseline differences could mask any true difference in the efficacy of remimazolam compared to dexmedetomidine or propofol. Furthermore, variations in study quality, including differences in blinding methodologies and outcome reporting, as well as the timing of POD assessment, may also have contributed to the observed lack of significant difference. Specifically, the definition of POD and its method of assessment varied across the included studies, with some using formal diagnostic tools (e.g., CAM) [20] and others not [21]. These methodological variations may have introduced a degree of bias and heterogeneity that impacted our ability to determine the true effect. Despite these limitations, the finding that remimazolam was associated with fewer hypotensive events when compared to dexmedetomidine and propofol highlights a potential clinical advantage for patients at risk for hypotension.

In response to possible heterogeneity in the study, we believe that it stems from four main sources: first, study protocol variations, studies differed in remimazolam administration: some used boluses [11], others continuous infusions [23], and initiation timing varied [24]. These protocol variations may have affected remimazolam’s effectiveness; second, patient characteristics, studies included different populations: some focused on elderly with comorbidities [14, 20], others included pediatrics [26] or a broader range of patients [22]. These differences can impact baseline POD risk and response to anesthesia; third, remimazolam dosing variations, loading doses varied (2.5–5 mg/kg) and infusion rates varied (0.2–1.0 mg/kg/h). Some studies used lower doses for shorter procedures [23], others higher in older patients [11, 20]. Dosing variability may have contributed to differences in efficacy and adverse events, and concurrent use of other medications further adds to this heterogeneity. The variability across studies limits our ability to draw firm conclusions. Our findings highlight the need for standardized protocols and studies with similar populations to better understand remimazolam’s effect. Fourth, differences in surgical procedures likely influenced the observed heterogeneity. The diverse range of surgical types and their varying levels of complexity introduced differing degrees of physiological stress and inflammation, potentially impacting the study results. The substantial variability across these study and patient characteristics limits our ability to draw definitive conclusions from the meta-analysis. Therefore, our findings underscore the importance of implementing standardized protocols and focusing on more homogeneous patient populations in future research to better elucidate the effect of remimazolam on POD.

Existing systematic reviews and meta-analyses have explored the effect of other sedatives on POD. For example, several meta-analyses have shown a reduction in POD incidence with dexmedetomidine compared to placebo or other sedatives [41–43], which may account for the lack of a statistically significant difference between remimazolam and dexmedetomidine in our findings. However, these studies often do not report the hemodynamic changes with these anesthetics, making a direct comparison difficult. Propofol, while widely used, has shown inconsistent effects on POD incidence in different reviews [44], further highlighting the need for more focused studies and comparisons.

A demonstrably safe margin between the onset of unconsciousness and respiratory depression is observed across all age demographics. Remimazolam can be administered safely without inducing significant hemodynamic instability [45]. Importantly, this study did show a significantly lower risk of intraoperative hypotension with remimazolam compared to both dexmedetomidine and propofol. This hemodynamic stability is a clinically relevant benefit, particularly for patients at increased risk of hypotension, such as the elderly, or those with cardiac conditions. This could potentially reduce the need for vasopressors, and the associated costs and risks of this clinical intervention. Therefore, remimazolam may represent a better choice in patients where maintaining hemodynamic stability is a primary concern. This is an important advantage over propofol which can be associated with hypotension [46]. While dexmedetomidine also can provide hemodynamic stability, remimazolam has demonstrated less respiratory depression and quicker awakening times. In clinical practice, individualized dosage adjustments should be made based on patient characteristics and age, with careful consideration given to choosing a sedative that aligns with surgical requirements and economic factors.

This meta-analysis employed a standardized approach to directly compare predefined risk exposures and primary outcomes. However, there are several limitations to this study: (1) the trial had limited enrollment in some subgroups; (2) it was not feasible to standardize all anesthetic variables outside of the control and experimental groups, as these variables may change during the perioperative period; (3) meta-analysis of the effects across disparate patient subgroups was not attainable; (4) the study included patients across a wide age range, and age-related physiological changes may affect the efficacy and safety of different anesthetic agents; (5) while the Cochrane risk of bias assessment suggested a generally low risk of bias, some study had unclear methods for blinding [14], potentially introducing bias in outcome assessment; and (6) there was some variation in the definition and reporting of POD across studies, which may have contributed to heterogeneity, specifically, the timing of POD assessment varied across the included studies, and some studies may not have employed the same rigorous diagnostic criteria.

## Conclusion

In conclusion, perioperative administration of remimazolam reduced the POD risk in patients undergoing non-cardiac surgery, and there was no significant difference in the ability of remimazolam compared to dexmedetomidine or propofol to reduce POD in patients in the category. However, remimazolam exhibited a notable decrease in the occurrence of intraoperative hypotension. Perioperative management with effective individualized sedation remains the key to preventing POD.

## Data Availability

No datasets were generated or analysed during the current study.
